# Glycemic burden-adjusted urinary glucose excretion deficit is associated with hyperuricemia in patients with type 2 diabetes

**DOI:** 10.3389/fendo.2026.1939076

**Published:** 2026-09-18

**Authors:** Liying Wang, Yun Wang, Ying Gong, Houfa Geng

**Affiliations:** 1 Department of Endocrinology, Xuzhou Central Hospital, Southeast University, Xuzhou, Jiangsu, China; 2 Department of Nephrology, The Affiliated Huaian No.1 People’s Hospital of Nanjing Medical University, Huai’an, Jiangsu, China

**Keywords:** glycemic burden, hyperuricemia, glycemic burden‑adjusted urinary glucose excretion deficit, serum uric acid, type 2 diabetes mellitus, urinary glucose excretion

## Abstract

**Objective:**

To investigate the associations of glycemic burden-adjusted urinary glucose excretion deficit (GB-UGE deficit) with serum uric acid levels and prevalent hyperuricemia in patients with type 2 diabetes mellitus (T2DM).

**Methods:**

A total of 887 patients with T2DM were enrolled. GB-UGE deficit was defined as the inverse standardized residual from a linear regression of ln(urinary glucose excretion + 1) on glycated hemoglobin, fasting glucose, 2-hour postprandial glucose and eGFR; a higher GB-UGE deficit indicates less urinary glucose excretion than expected for a given glycemic burden. Participants were grouped by hyperuricemia status, with baseline demographic, metabolic and renal data collected. Statistical analyses included multiple linear regression, quartile-based logistic regression, stepwise-adjusted logistic regression, restricted cubic splines (RCS), ROC curves and sensitivity analyses.

**Results:**

Patients with hyperuricemia had a higher GB-UGE deficit (*P < 0.001*). In the fully adjusted linear model, each 1-SD higher GB-UGE deficit was associated with a 15.44umol/L higher serum uric acid concentration (95% CI: 9.89 to 20.98,*P < 0.001*). RCS analysis demonstrated an overall association between GB-UGE deficit and serum uric acid (*P for overall association < 0.0001*), with borderline evidence of nonlinearity (*P for nonlinearity = 0.0479*); the association with prevalent hyperuricemia was approximately linear. Quartile analyses showed progressively higher odds of prevalent hyperuricemia (*P for trend < 0.001*), and the sensitivity analyses produced estimates in the same direction. In the exploratory ROC analysis, the AUC increased by 0.021 from 0.787 for the conventional model to 0.808 for the model containing GB-UGE deficit (*DeLong P = 0.0014*); however, the AUC increment between the UGE-containing model and the GB-UGE deficit model was merely 0.003 and lacked statistical significance.

**Conclusion:**

In this single-center cross-sectional study of hospitalized T2DM patients, GB-UGE deficit was associated with elevated serum uric acid and prevalent hyperuricemia. As an exploratory cohort-specific residual index rather than a validated clinical marker, it may signal suboptimal urinary glucose excretion, with hypothetical links to disturbed renal tubular glucose and urate co-transport. Prospective multicenter external cohorts are required to verify its reproducibility, biological mechanism and translational value.

## 1 Introduction

Hyperuricemia (HUA) is a metabolic disorder related to disturbed purine metabolism and is strongly associated with hypertension, insulin resistance, type 2 diabetes mellitus (T2DM), kidney disorders, and cardiovascular and cerebrovascular diseases (1–4). In patients with T2DM, HUA has been linked to diabetic microvascular complications, adverse cardiovascular outcomes, and all-cause mortality (5–10). Therefore, identifying metabolic characteristics and modifiable factors associated with serum uric acid in T2DM may help improve integrated disease management and long-term prognosis. Collectively, co-management of serum uric acid and blood glucose has substantial clinical significance for people living with T2DM.

The kidneys regulate systemic glucose homeostasis through endogenous gluconeogenesis and reabsorption of filtered glucose within the proximal tubules (11). Inhibition of renal tubular glucose reabsorption to enhance urinary glucose excretion has emerged as an effective therapeutic strategy for diabetes mellitus. Clinical studies have shown that sodium-glucose cotransporter 2 (SGLT2) inhibitors can reduce serum uric acid levels while exerting glucose-lowering effects (4), and accumulating evidence attributes this urate-lowering effect partly to increased urinary glucose excretion (12, 13). Given these observations, the relationship between urinary glucose excretion and hyperuricemia warrants further study. However, readily available measures that characterize renal glucose handling under comparable glycemic exposure remain limited; any residual-based measure requires careful validation before clinical use.

Urinary glucose excretion is influenced by multiple factors, including glycated hemoglobin, fasting plasma glucose, postprandial glucose, and renal function. Simple analysis of absolute urinary glucose levels cannot distinguish urinary glucose driven by glycemic burden from interindividual variationin renal glucose handling. We therefore derived a glycemic burden-adjusted urinary glucose excretion deficit (GB-UGE deficit) as an exploratory within-cohort residual measure of the extent to which observed urinary glucose excretion was lower than predicted by HbA1c, fasting plasma glucose, 2-hour postprandial glucose, and eGFR. A higher value indicates less urinary glucose excretion than expected from this fitted model; it does not directly measure transporter activity or establish a clinical threshold.

Herein, this study aimed to evaluate the associations of GB-UGE deficit with serum uric acid concentrations and prevalent hyperuricemia among patients with T2DM.

## 2 Materials and methods

### 2.1 Study design and study subjects

This single-center cross-sectional study was approved by the Medical Ethics Committee of Xuzhou Central Hospital, Southeast University (Ethics Approval No.: XZXY-LJ-20200902-035). All study procedures were conducted in accordance with the principles outlined in the Declaration of Helsinki, and written informed consent was obtained from all enrolled patients prior to participation.

A total of 887 patients with T2DM hospitalized at Xuzhou Central Hospital, Southeast University between 2022 and 2024 were consecutively enrolled in the present study. The diagnosis of T2DM was established based on the 1999 World Health Organization (WHO) criteria: fasting plasma glucose (FPG) >= 7.0 mmol/L or 2-hour postprandial plasma glucose (2hPG) >= 11.1 mmol/L. The primary outcome was prevalent hyperuricemia, defined as serum uric acid >= 420 μmol/L in men and >= 360 μmol/L in women. A sensitivity analysis used a universal threshold of >= 420 μmol/L.

Exclusion criteria were as follows: previous history of gout, chronic alcohol abuse, anemia, malignant tumors, chronic glomerulonephritis, liver dysfunction, acute diabetic complications, severe cardiovascular and cerebrovascular diseases, acute kidney injury, pancreatitis, active infection, malignant hematological diseases, or patients receiving maintenance dialysis. Additional exclusions included subjects who had taken urate-lowering agents or medications interfering with uric acid metabolism within the preceding one month (including allopurinol, benzbromarone, aspirin, diuretics, losartan, pyrazinamide, cyclosporine, azathioprine, etc.), patients treated with SGLT2 inhibitors, individuals with incomplete medical records, and those missing core study variables.

### 2.2 Clinical and laboratory variables

#### 2.2.1 Demographic and clinical data

Basic information including sex, age, and diabetes duration was recorded. Height, body weight, and blood pressure were measured systematically. Height and body weight were measured with participants wearing light clothing and barefoot, and body mass index (BMI, kg/m²) was subsequently calculated. After a 10-minute resting period, systolic blood pressure (SBP) and diastolic blood pressure (DBP) on the right upper arm were measured three times using a mercury sphygmomanometer, and the average value was adopted for statistical analyses.

#### 2.2.2 Biochemical indices

All participants underwent an 8–12 h overnight fast before venous blood samples were collected from the cubital vein the next morning. An oral glucose tolerance test (OGTT) was performed to detect 2-hour postprandial serum insulin, serum C-peptide, and 2hPG levels. Concentrations of plasma glucose, serum uric acid (SUA), serum creatinine (Scr), triglycerides (TG), total cholesterol (TC), high-density lipoprotein cholesterol (HDL-C), and low-density lipoprotein cholesterol (LDL-C) were quantified using an automatic biochemical analyzer (Model 7600, Hitachi, Japan). Glycated hemoglobin A1c (HbA1c) was determined via high-performance liquid chromatography (HLC-723G7 HbA1c analyzer, Tosoh, Japan). Serum insulin and C-peptide levels were measured using radioimmunoassays (Pharmacia, USA). The homeostasis model assessment of insulin resistance (HOMA-IR) was calculated using the formula: HOMA-IR = (FPG × fasting insulin)/22.5. The estimated glomerular filtration rate (eGFR) was calculated using the following formula (14): eGFR = 175×Scr^-1.234^×age^-0.179^× 0.79. For men: eGFR = 175×Scr^-1.234^× age^-0.179^. For 24-hour urinary glucose excretion (UGE) quantification, urine was collected from 7:00 a.m. on day 2 to 7:00 a.m. on day 3 of hospitalization. The first voided urine sample at 7:00 a.m. on day 2 was discarded, and the total mixed 24-hour urine specimen was submitted for testing. Urinary glucose concentration was detected using an automatic biochemical analyzer (Model 7600, Hitachi, Japan).Total 24-hour UGE (g/day) was calculated as urinary glucose concentration multiplied by the total collected 24-hour urine volume.

### 2.3 Construction of GB-UGE deficit

Because 24-hour urinary glucose excretion was right-skewed, it was transformed as ln(UGE + 1). A linear regression model was then fitted with ln(UGE + 1) as the dependent variable and HbA1c, fasting plasma glucose, 2-hour postprandial glucose, and eGFR as predictors. The residual from this model represented the difference between observed and expected urinary glucose excretion. The inverse residual was defined as GB-UGE deficit and standardized to a z score. Thus, a higher GB-UGE deficit indicates lower urinary glucose excretion than expected for the observed glycemic burden. This primary derivation model was specified to capture core glycemic and renal determinants of UGE rather than to function as a diagnostic model. Age, sex, and BMI were included in an alternative residual model in a sensitivity analysis. No clinical cutoff was prespecified or validated. The derivation model was refitted in the full analytic cohort of 887 participants. The fitted intercept and regression coefficients, together with their standard errors, 95% confidence intervals, and P values, are reported in **Supplementary Table 1**. Model fit was summarized using R², adjusted R², root mean squared error (RMSE), and the overall F statistic. For each participant, the unstandardized GB-UGE deficit was calculated as predicted minus observed ln(UGE + 1), equivalent to the inverse regression residual, and was subsequently standardized using its cohort mean and standard deviation.

Formula used in this analysis:


ln(UGE + 1)=β0+β1×HbA1c+β2×FPG+β3×2hPG+β4×eGFR+ϵ

GB−UGE deficit=−ϵ;standardized GB−UGE deficit=(GB−UGE deficit − mean)/SD.

### 2.4 Statistical analysis

All statistical analyses were performed using R software (version 4.5.2). Normally distributed continuous variables were reported as mean ± standard deviation, while skewed continuous variables were presented as median (interquartile range). Categorical variables were summarized as counts (percentages). Between-group comparisons were performed using independent samples t-tests, Mann-Whitney U tests, or χ² tests according to data distribution.

Statistical approaches comprised multiple linear regression, quartile-stratified logistic regression, hierarchically adjusted logistic regression, restricted cubic splines (RCS), receiver operating characteristic (ROC) curve analysis, and sensitivity analysis. Multiple linear regression was used to estimate the adjusted association between standardized GB-UGE deficit and SUA. Hierarchical logistic regression models were constructed to estimate the association between GB-UGE deficit and prevalent hyperuricemia at three adjustment levels: Model 1 adjusted for age and sex; Model 2 additionally adjusted for diabetes duration, BMI, SBP, DBP, HbA1c, FPG, 2hPG, and eGFR; and Model 3 additionally adjusted for HOMA-IR, triglycerides, HDL-C, LDL-C, and Scr. For each RCS analysis, four knots were placed at the 5th, 35th, 65th, and 95th percentiles. The overall association was assessed by a joint Wald test of all spline terms, and nonlinearity was assessed by a joint Wald test of the nonlinear spline terms.

GB-UGE deficit was categorized into empirical quartiles for descriptive dose-response analyses, with the lowest quartile as the reference. These cohort-specific quartiles were not intended as clinical cutoffs. RCS models were used to characterize the continuous shape of the associations and to evaluate whether the data supported an obvious threshold pattern.

Two sensitivity analyses assessed whether the association materially changed when hyperuricemia was uniformly defined as SUA ≥ 420 μmol/L and when an alternative GB-UGE deficit was derived with additional adjustment for age, sex, and BMI. ROC curves and AUC values were compared for three exploratory, internally evaluated models: a conventional clinical model, that model plus UGE, and that model plus GB-UGE deficit. The ROC analysis was not intended to establish a diagnostic tool, and no externally validated cutoff, calibration analysis, or decision-curve analysis was available. All statistical tests were two-tailed, and P < 0.05 was considered statistically significant.

## 3 Results

The GB-UGE derivation model included all 887 participants and explained 30.3% of the variation in ln(UGE + 1) (R² = 0.3035; adjusted R² = 0.3003; RMSE = 1.1353; F[4,882] = 96.07, *P < 0.001*). Complete fitted coefficients are provided in **Supplementary Table 1**.

### 3.1 Baseline clinical characteristics of study participants

A total of 887 patients with T2DM were enrolled in the present study, including 273 patients with hyperuricemia (HUA, 30.8%) and 614 patients without HUA (non-HUA, 69.2%). Baseline demographic, metabolic, and renal parameters of all participants are summarized in **Table 1**. Significant intergroup differences were observed in BMI, UGE, HOMA-IR, TG, HDL-C, Scr, eGFR, SUA, and GB-UGE deficit (*P < 0.05*). No significant between-group differences were detected in age, sex distribution, diabetes duration, systolic blood pressure, diastolic blood pressure, HbA1c, FPG,LDL-C or 2hPG (*P > 0.05*).

**Table 1:** Baseline characteristics by hyperuricemia status.

Variable	Overall (n=887)	Non-HUA (n=614)	HUA (n=273)	P value
Age, years	58.12 (13.10)	57.95 (12.34)	58.51 (14.68)	0.557
Male, n (%)	544 (61.3)	388 (63.2)	156 (57.1)	0.102
Diabetes duration, years	8.00 [2.00, 14.00]	8.00 [2.00, 14.00]	8.00 [2.00, 15.00]	0.848
BMI, kg/m²	25.44 (3.82)	24.84 (3.37)	26.78 (4.39)	<0.001
SBP, mmHg	131.83 (15.36)	131.46 (15.40)	132.67 (15.27)	0.277
DBP, mmHg	76.22 (10.14)	76.25 (10.00)	76.14 (10.48)	0.88
UGE, g/day	7.79 [0.66, 22.68]	9.43 [1.16, 24.12]	4.86 [0.31, 19.55]	<0.001
HbA1c, %	9.02 (2.32)	9.05 (2.24)	8.94 (2.48)	0.484
FPG, mmol/L	9.21 (3.51)	9.13 (3.39)	9.37 (3.78)	0.351
2hPG, mmol/L	15.57 (5.46)	15.56 (5.49)	15.58 (5.42)	0.974
HOMA-IR	2.57 [1.21, 4.65]	2.16 [1.12, 4.06]	3.66 [1.84, 6.12]	<0.001
TG, mmol/L	1.74 [1.25, 2.62]	1.58 [1.15, 2.33]	2.13 [1.54, 4.38]	<0.001
HDL-C, mmol/L	1.21 (0.44)	1.24 (0.49)	1.13 (0.26)	0.001
LDL-C, mmol/L	2.91(0.90)	2.91(0.88)	2.91 (0.96)	0.988
Creatinine, μmol/L	55.20 [45.10, 67.00]	52.40 [43.40, 62.48]	62.00 [50.40, 76.70]	<0.001
eGFR, mL/min/1.73 m²	135.67 [108.75, 169.36]	143.64 [118.04, 178.66]	119.65 [90.97, 150.53]	<0.001
SUA, μmol/L	336.46 (96.77)	285.81 (61.69)	450.39 (55.73)	<0.001
GB-UGE deficit	0.00 (1.00)	−0.11 (1.01)	0.25 (0.92)	<0.001

Total participants n=887, HUA n=273 (30.8%), non-HUA n=614 (69.2%); Data are presented as mean (SD), median [interquartile range], or n (%), as appropriate. HUA, hyperuricemia; non-HUA, without hyperuricemia; BMI, body mass index; SBP, systolic blood pressure; DBP, diastolic blood pressure; UGE, urinary glucose excretion; HbA1c, glycated hemoglobin; FPG, fasting plasma glucose; 2hPG, 2-hour postprandial plasma glucose; HOMA-IR, homeostasis model assessment of insulin resistance; TG, triglycerides; HDL-C, high-density lipoprotein cholesterol; LDL-C, low-density lipoprotein cholesterol; SUA, serum uric acid; eGFR, estimated glomerular filtration rate; GB-UGE deficit, glycemic burden-adjusted urinary glucose excretion deficit.

Compared with the non-HUA group, patients with HUA exhibited higher BMI, HOMA-IR, TG, Scr, SUA, and GB-UGE deficit, alongside lower HDL-C, UGE, and eGFR levels. The mean GB-UGE deficit was 0.25 in the HUA group versus -0.11 in the non-HUA group (*P < 0.001*), indicating relatively lower urinary glucose excretion than expected for the observed glycemic burden among individuals with hyperuricemia.

### 3.2 Linear and nonlinear associations between GB-UGE deficit and serum uric acid

#### 3.2.1 Multiple linear regression analysis

Multiple linear regression showed a positive adjusted association between GB-UGE deficit and SUA. After adjustment for age and sex (Model 1), each 1-SD increment in GB-UGE deficit corresponded to a 15.86 μmol/L higher SUA concentration (95% CI: 9.44 to 22.29, *P < 0.001*). Further adjustment for diabetes duration, BMI, blood pressure, glycemic variables, and eGFR (Model 2) yielded a similar estimate. In Model 3, which additionally included insulin resistance, lipid variables, and serum creatinine, the estimate was 15.44 μmol/L (95% CI: 9.89 to 20.98,*P < 0.001*). These results indicate that the observed association persisted after adjustment for the measured covariates; they do not exclude residual confounding or establish causality (**Table 2**).

**Table 2:** Association of GB-UGE deficit with serum uric acid.

Model	β coefficient	95% CI	P value
Model 1	15.86	9.44–22.29	<0.001
Model 2	15.06	9.13–20.99	<0.001
Model 3	15.44	9.89–20.98	<0.001

Model 1 adjusted for age and sex. Model 2 adjusted for Model 1 plus diabetes duration, BMI, systolic blood pressure, diastolic blood pressure, HbA1c, fasting plasma glucose, 2-hour postprandial plasma glucose, and eGFR. Model 3 adjusted for Model 2 plus HOMA-IR, triglycerides, HDL-C, LDL-C, and serum creatinine. β coefficients represent the change in serum uric acid (μmol/L) per 1-SD increment in GB-UGE deficit.

#### 3.2.2 Restricted cubic spline characterization of the continuous association

A fully adjusted 4-knot RCS model was used to characterize the continuous association between GB-UGE deficit and SUA (**Figure 1**). The overall association was statistically significant (F[3,867] = 12.03, *P < 0.0001*), while the formal test provided borderline evidence of nonlinearity (F[2,867] = 3.05, *P = 0.0479*). The fitted curve showed modest curvature, with lower predicted SUA around a standardized GB-UGE deficit of approximately -1 and higher predicted values around 1, followed by slight attenuation at higher values. Given substantially widened confidence intervals at both extremes of the GB-UGE-deficit distribution, statistical precision is reduced in these regions. This nonlinear distribution may suggest that the correlation between impaired tubular glucose excretion and serum uric acid levels differs at distinct GB-UGE deficit levels, providing novel observational evidence for heterogeneous glucose-urate tubular interplay among T2DM patients. Collectively, these observations provide suggestive evidence for a non-monotonic association between GB-UGE deficit and serum uric acid, yet they are insufficient to confirm a robust nonlinear relationship, identify a meaningful clinical threshold, or establish the underlying biological mechanisms.

**Figure 1:**
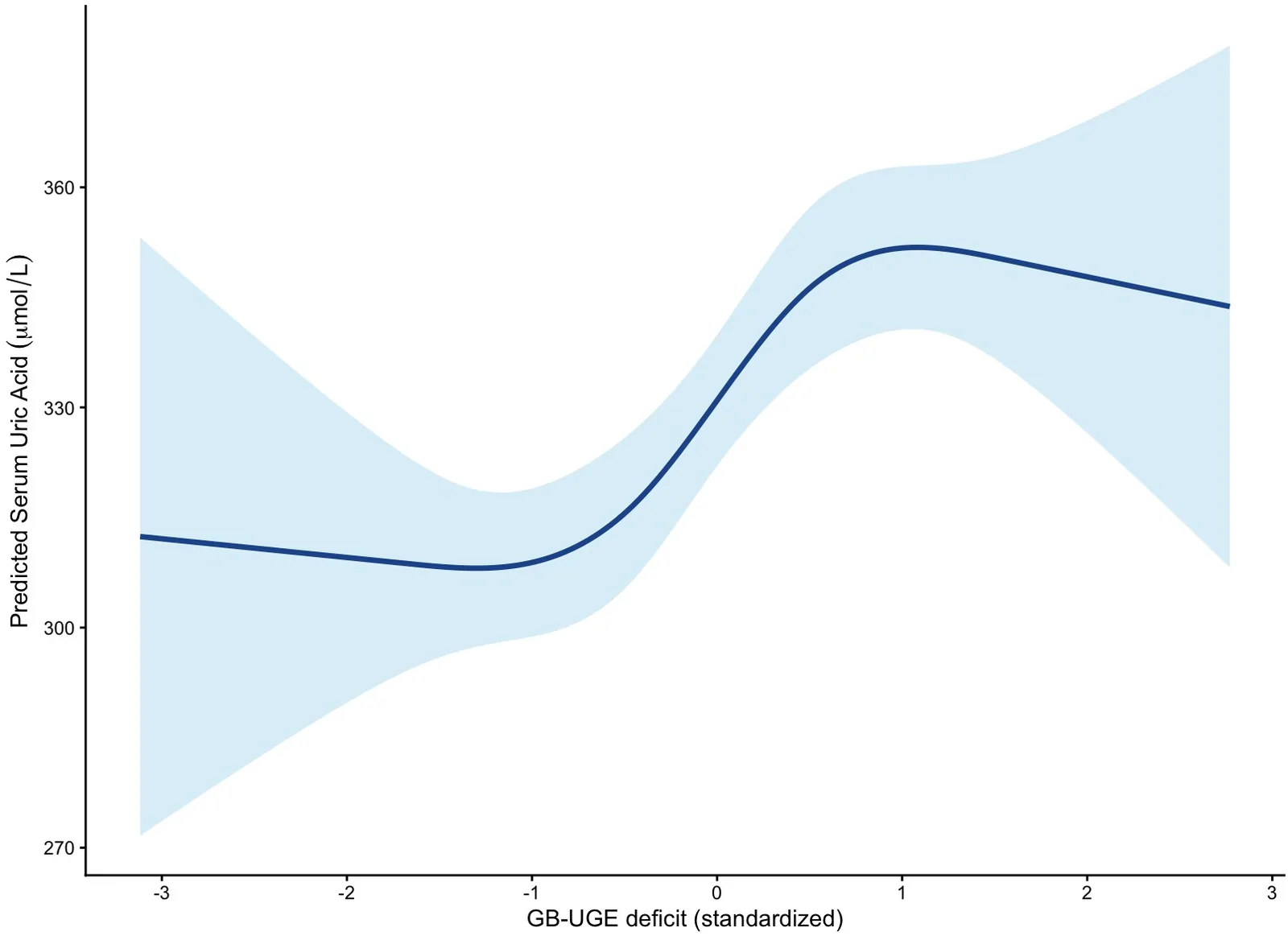
Restricted cubic spline (RCS) curve showing the cross-sectional association between standardized GB-UGE deficit and serum uric acid (SUA, μmol/L). Analyses relied on a 4-knot spline model fully adjusted for age, diabetes duration, BMI, blood pressure, glycemic markers, eGFR, lipid profiles, insulin resistance and serum creatinine. Shaded bands denote 95% confidence intervals. P for overall association < 0.0001; P for nonlinearity = 0.0479.

### 3.3 Quartile stratification of GB-UGE deficit and dose-response trends

Stratification by GB-UGE deficit quartiles revealed an overall upward pattern in SUA levels and hyperuricemia prevalence across ascending quartiles. Participants were divided into four quartile groups (Q1-Q4, 221–222 subjects per stratum). The prevalence of HUA was 20.3%, 28.4%, 29.9%, and 44.6% from Q1 to Q4, with corresponding mean SUA concentrations of 318.0, 336.0, 335.0, and 358.0 μmol/L (**Figure 2**; **Table 3**).The quartile boundaries were derived from this cohort and should not be interpreted as clinical decision thresholds.

**Figure 2:**
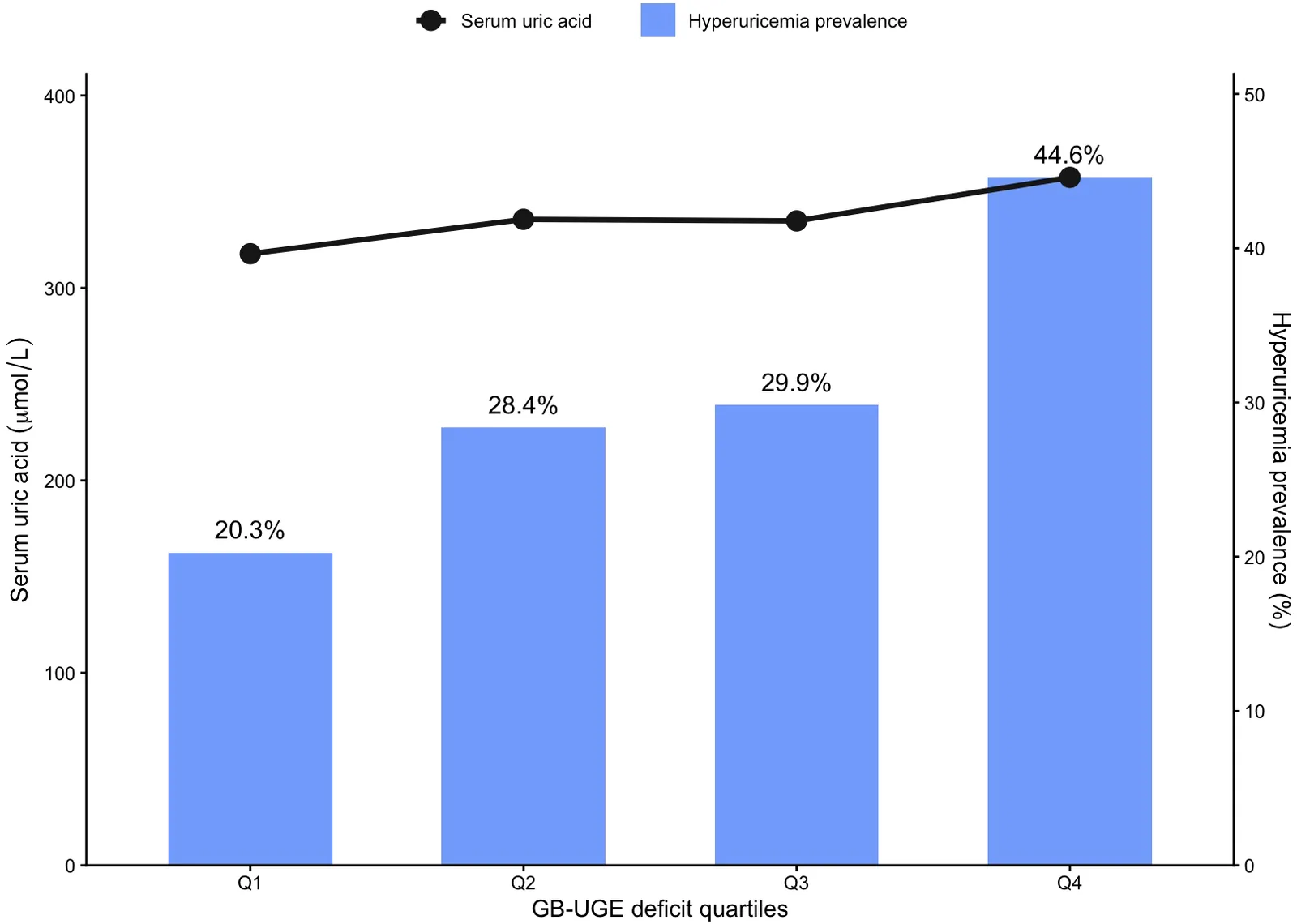
Mean serum uric acid concentration and prevalence of hyperuricemia across four empirical GB-UGE deficit quartiles (Q1–Q4). N per quartile: Q1 = 222, Q2 = 222, Q3 = 221, Q4 = 222.

**Table 3:** Quartiles of GB-UGE deficit and odds of prevalent hyperuricemia.

Quartile	n	Mean SUA, μmol/L	HUA, n (%)	Adjusted OR (vs Q1)	95% CI	P value
Q1	222	318.0	45 (20.3)	1.00	Reference	–
Q2	222	336.0	63 (28.4)	1.81	1.08–3.04	0.026
Q3	221	335.0	66 (29.9)	2.22	1.31–3.75	0.003
Q4	222	358.0	99 (44.6)	3.80	2.31 –6.25	<0.001
P for trend						<0.001

Odds ratios (ORs) and 95% CIs were derived from fully adjusted logistic regression models with the lowest quartile (Q1) set as the reference group. Linear trend across quartiles was tested. Mean serum uric acid and hyperuricemia prevalence are presented for each GB-UGE deficit stratum. P for trend was calculated by treating quartiles as an ordered categorical variable (assigned scores 1, 2, 3, 4 for Q1–Q4) in the fully adjusted multivariable logistic regression model.

Fully adjusted logistic regression with Q1 as the reference demonstrated higher odds of prevalent HUA across higher strata: Q2 was associated with higher odds of hyperuricemia (OR = 1.81, 95% CI: 1.08 to 3.04, *P = 0.026*); Q3 showed a further increase (OR = 2.22, 95% CI: 1.31 to 3.75, *P = 0.003*); and Q4 was associated with 3.80-fold higher odds of hyperuricemia relative to Q1 (OR = 3.80, 95% CI: 2.31 to 6.25, *P < 0.001*). A significant linear trend was observed across quartiles (*P for trend < 0.001*), supporting a graded positive association between GB-UGE deficit and prevalent hyperuricemia.

### 3.4 Adjusted logistic regression and sensitivity analyses of prevalent hyperuricemia

Hierarchically adjusted logistic regression models were used to estimate the association between standardized GB-UGE deficit and prevalent hyperuricemia; the estimates are summarized in **Figure 3**. In Model 1, each 1-SD increment in GB-UGE deficit was associated with an OR of 1.43 (95% CI: 1.23 to 1.67). The corresponding OR was 1.47 (95% CI: 1.24 to 1.75) in Model 2 and 1.53 (95% CI: 1.28 to 1.84) in Model 3.Thus, the association remained statistically significant after adjustment for the measured demographic, metabolic, and renal covariates. Because the design was cross-sectional and some relevant exposures were not measured, these estimates should not be interpreted as causal effects.

**Figure 3:**
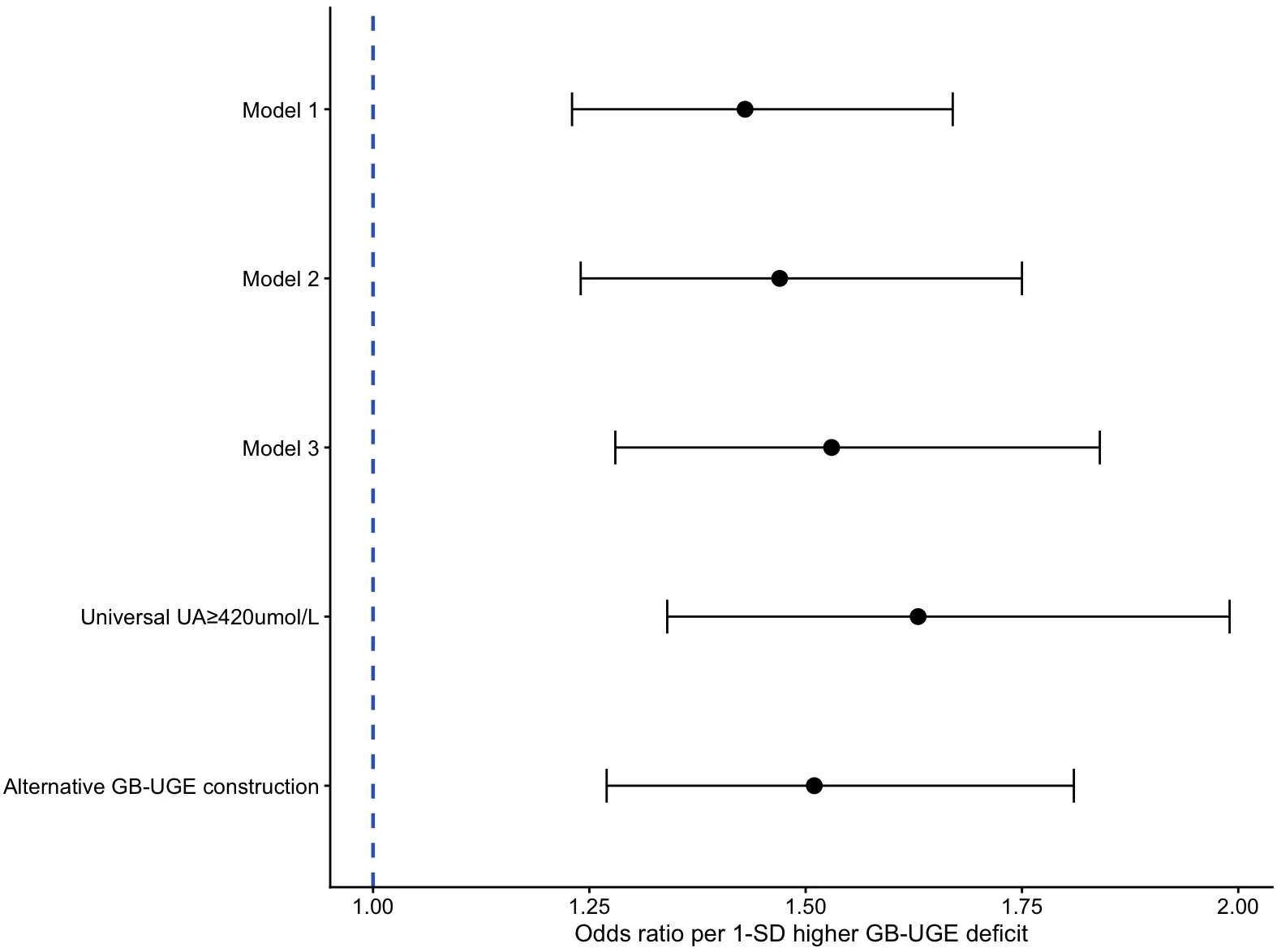
Fully adjusted odds ratios (ORs) and 95% CIs for prevalent hyperuricemia per 1-SD higher GB-UGE deficit across three hierarchical adjustment models. Model 1 adjusted for age and sex. Model 2 adjusted for Model 1 plus diabetes duration, BMI, systolic blood pressure, diastolic blood pressure, HbA1c, fasting plasma glucose, 2-hour postprandial plasma glucose, and eGFR. Model 3 adjusted for Model 2 plus HOMA-IR, triglycerides, HDL-C, LDL-C, and creatinine. ORs represent the odds of hyperuricemia per 1-SD increment in GB-UGE deficit.

In sensitivity analyses, use of a universal SUA threshold of ≥ 420 μmol/L yielded an OR of 1.63(95% CI: 1.34 to 1.99), and use of an alternative residual metric additionally adjusted for age, sex, and BMI yielded an OR of 1.51 (95% CI: 1.27 to 1.81). The direction of association was unchanged in these analyses, but this internal consistency does not substitute for repeated measurement or external validation (**Figure 3**).

### 3.5 Restricted cubic spline analysis of prevalent hyperuricemia probability

A fully adjusted 4-knot RCS model was used to visualize the continuous association between GB-UGE deficit and the predicted probability of prevalent hyperuricemia (**Figure 4**). The overall Wald test was significant (χ² = 23.35,*P < 0.0001*), whereas the nonlinearity test was not (χ² = 0.11, *P = 0.9474*). The fitted relationship was therefore compatible with an approximately linear increase over the observed range, with no statistically supported inflection point or threshold. Accordingly, these data do not provide a clinically meaningful GB-UGE deficit cutoff.

**Figure 4:**
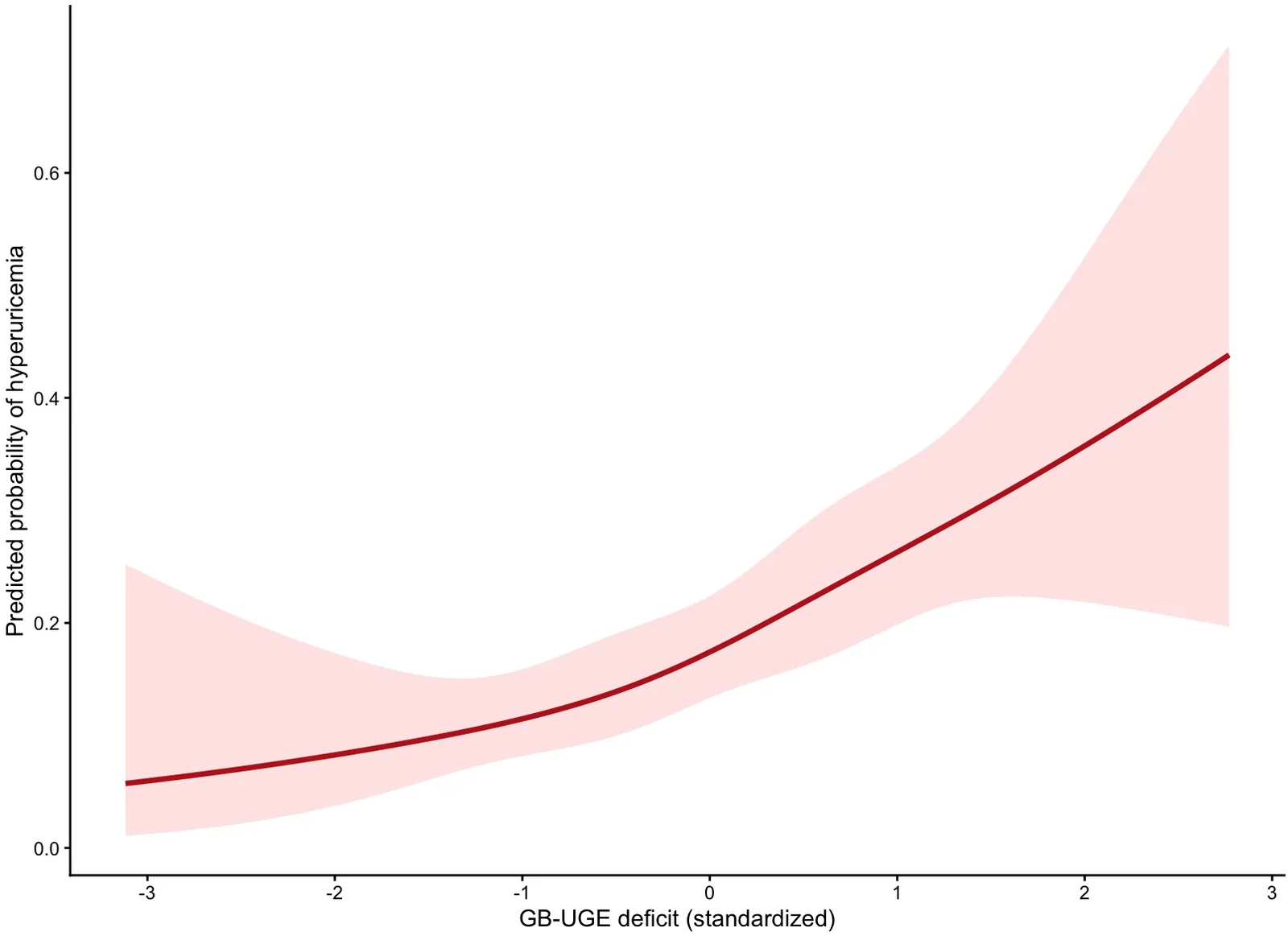
Restricted cubic spline curve illustrating the cross-sectional association between standardized GB-UGE deficit and the probability of prevalent hyperuricemia, constructed using a fully adjusted 4-knot spline model. P for overall association < 0.0001; P for nonlinearity = 0.9474. Shaded bands denote 95% CIs.

### 3.6 Exploratory comparison of model discrimination

Using SUA ≥ 420 μmol/L as the uniform definition of hyperuricemia, three internally evaluated models were compared (**Figure 5**). The AUC was 0.787 for the conventional model (Model 1), 0.805 after adding 24-hour UGE (Model 2), and 0.808 after adding GB-UGE deficit (Model 3). Compared with Model 1, the absolute AUC increase was 0.018 for Model 2 (DeLong P = 0.0021) and 0.021 for Model 3 (DeLong P = 0.0014). The 0.003 AUC difference between Models 2 and 3 was not statistically significant(DeLong P = 0.3852). Thus, Model 3 showed only modest improvement over conventional factors, and the specific incremental value of GB-UGE deficit beyond UGE was not established. Without external validation, calibration assessment, or decision-curve analysis, these findings do not demonstrate clinical utility.

**Figure 5:**
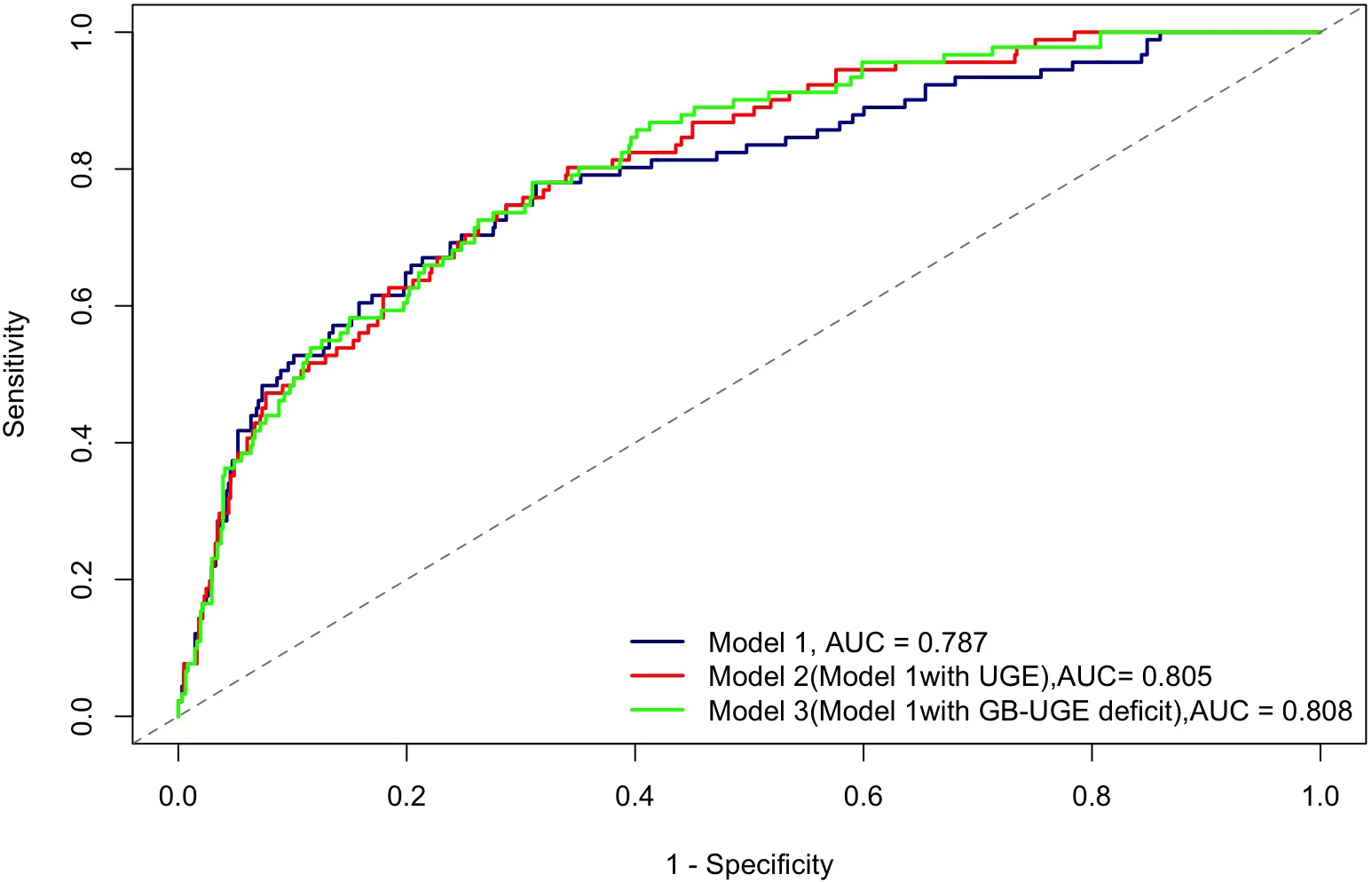
Exploratory ROC curves for discrimination of prevalent hyperuricemia within the study sample. Model 1 represents the conventional clinical model; Model 2 adds UGE; Model 3 adds GB-UGE deficit. These internally evaluated ROC comparisons were exploratory and do not establish a clinically useful prediction model. ROC, receiver operating characteristic; UGE, urinary glucose excretion.

## 4 Discussion

In this single-center cross-sectional study of 887 hospitalized patients with T2DM, GB-UGE deficit was associated with higher SUA and higher odds of prevalent hyperuricemia after adjustment for measured covariates. The SUA spline showed an overall association and borderline evidence of nonlinearity (P for nonlinearity = 0.0479), whereas the association with prevalent hyperuricemia was approximately linear. Sensitivity analyses yielded estimates in the same direction. The exploratory ROC analysis revealed only modest AUC improvement relative to the conventional model; furthermore, we could not formally assess whether GB-UGE deficit provides additional discriminative value beyond UGE. Our findings only put forward exploratory hypotheses regarding glucose-urate tubular metabolism, and sufficient external evidence is still required to support its causal implication and clinical utility.

Consistent with previous clinical evidence, UGE was negatively correlated with serum uric acid after adjustment for confounders in patients with newly diagnosed T2DM, and individuals with higher UGE exhibited a lower likelihood of hyperuricemia (12). Additional studies focusing on urinary glucose and urate excretion demonstrated that SUA did not rise continuously with increasing FPG; once FPG exceeded a critical threshold that triggered glucosuria, urinary urate excretion was further augmented, accompanied by a subsequent decline in SUA concentrations (15). A higher urinary glucose excretion rate was positively correlated with urinary urate excretion and renal urate clearance, while being inversely associated with SUA levels; consistently elevated urinary glucose excretion was also associated with reduced hyperuricemia in patients with T2DM (16). Our findings are in line with these prior observations and further suggest a non-monotonic relationship between GB-UGE deficit and SUA. However, given the wider confidence intervals at the extremes of the spline curve, this pattern should be interpreted cautiously and validated in external cohorts. Our findings are broadly consistent with these previous observations. Although the fitted SUA spline displayed modest curvature and the formal test provided borderline evidence of nonlinearity, the present data do not establish a reproducible non-monotonic relationship or a clinically meaningful threshold. This shape should therefore be interpreted cautiously and evaluated in independent cohorts.

From a biological mechanistic perspective, existing research has indicated that elevated urinary glucose excretion modulates renal urate transport and promotes urate elimination by altering intratubular glucose concentrations (17). Humans lack uricase, which is required to convert uric acid into soluble allantoin, and uric acid is therefore predominantly eliminated via urinary excretion. Approximately 90% of circulating uric acid undergoes reabsorption and secretion within the proximal renal tubules, with the remaining fraction excreted via the intestinal tract (18). Urate reabsorption is mediated by multiple apical anion transporters, including urate transporter 1 (URAT1), glucose transporter 9 isoform 2 (GLUT9v2), organic anion transporter 4 (OAT4), and organic anion transporter 10 (OAT10) (19–23). Apically expressed GLUT9v2 mediates counter-transport exchange between urinary glucose and urate along a concentration gradient (24), and it is widely recognized as a molecular mediator underlying glycosuria-induced uricosuric effects (13, 20, 25, 26).

In the present study, urinary urate excretion, fractional urate excretion, and renal transporter expression were not measured; consequently, this mechanistic explanation remains speculative.

HUA is highly prevalent and pathophysiologically relevant in T2DM populations. The temporal sequence between HUA and T2DM is difficult to delineate because the two conditions are closely interconnected. Elevated SUA has been associated with the onset and progression of T2DM, whereas diabetes itself may predispose individuals to hyperuricemia. This interplay may be attributable in part to dysregulated renal tubular glucose and urate handling: chronic hyperglycemia and insulin resistance can influence tubular transporters such as URAT1 and GLUT9, enhancing urate reabsorption and elevating circulating urate levels, which may in turn contribute to diabetic vascular and renal complications (27–29). Diabetic nephropathy can also disrupt normal urinary urate excretion (30, 31), while diabetes-related oxidative stress may disturb purine metabolism and promote uric acid accumulation (32). Regarding the glucose-urate metabolic interplay, an observational study reported that T2DM patients with higher SUA quintiles presented paradoxically lower HbA1c levels (33). Prior work has also identified a bell-shaped association between HbA1c, fasting glucose, and SUA (34), implying heterogeneous hyperuricemia patterns across glycemic strata. In our RCS analysis, the predicted probability of prevalent hyperuricemia increased approximately linearly with GB-UGE deficit, without a statistically supported inflection point. This observation is compatible with a graded association but does not define a causal or clinical threshold. Lower-than-expected UGE may be consistent with variation in proximal tubular glucose handling; however, because urinary urate handling and transporter activity were not measured, this interpretation remains a hypothesis and alternative explanations, including residual confounding, cannot be excluded.

The analytical contribution of this study is the comparison of observed UGE with the value predicted from HbA1c, fasting plasma glucose, 2-hour postprandial glucose, and eGFR, rather than relying only on raw UGE. This residualization reframes the exposure as lower-than-predicted UGE under the fitted model. Nevertheless, GB-UGE deficit is a cohort-derived statistical construct and not a direct measurement of renal glucose reabsorption. The quartile pattern supports further study of this phenotype, but the sample-specific quartiles and residual values should not be used as clinical cutoffs without prospective external validation.

The present findings have exploratory rather than immediate clinical implications. GB-UGE deficit may help formulate hypotheses about heterogeneity in the relation between glycemic exposure, UGE, and SUA, but it cannot currently classify individual patients or guide treatment. Future studies should use repeated outpatient urine collections, directly measure urinary urate and fractional urate excretion, assess SGLT2, GLUT9, and URAT1 where feasible, and evaluate reproducibility, calibration, and clinical utility in geographically and ethnically diverse external cohorts and across renal-function strata.

Several limitations should be acknowledged. First, the cross-sectional design does not establish temporal sequence or causality; reverse causation and bidirectional metabolic relationships remain possible. Second, UGE was based on a single 24-hour collection during hospitalization and may have been affected by short-term diet, water intake, intravenous fluids, treatment changes, collection completeness, and acute metabolic variation; no repeat measurement was available to quantify within-person reliability. Third, GB-UGE deficit was derived and evaluated in the same single-center inpatient cohort. It has not been externally validated, and the cohort-specific residual distribution and quartiles do not provide a clinically meaningful cutoff or establish generalizability to outpatients, other geographic or ethnic populations, or renal-function subgroups. Fourth, although patients with chronic alcohol abuse and those receiving SGLT2 inhibitors, urate-lowering treatment, or several medications known to influence urate metabolism were excluded, detailed dietary purine intake, fructose-containing beverage intake, daily alcohol consumption, water intake, medication dose and duration, other medication exposures, and genetic factors were not comprehensively measured. Residual and unmeasured confounding therefore remains likely. Standardized food frequency questionnaires will be adopted to quantify purine, fructose, and alcohol exposure for full multivariable adjustment in subsequent prospective studies. Fifth, urinary urate excretion, fractional urate excretion, and renal expression or activity of SGLT2, GLUT9, URAT1, and related transporters were not assessed, so the proposed tubular mechanism cannot be directly substantiated. Finally, the ROC analysis was exploratory and internally evaluated: the absolute AUC gain was modest, the difference between the UGE model and the model containing GB-UGE deficit was not significant, and calibration, clinical utility, and external performance were not assessed. Future multi-center, prospective cohort studies and basic mechanistic experiments are warranted to validate our findings and elaborate the underlying biological mechanisms.

## 5 Conclusions

In this single-center cross-sectional cohort of hospitalized T2DM patients, GB-UGE deficit representing lower-than-predicted urinary glucose excretion correlated with higher serum uric acid and hyperuricemia risk. This exploratory residual phenotype provides new observational evidence connecting aberrant tubular glucose excretion to urate metabolic disturbance, though it cannot yet serve as a validated physiological or clinical biomarker. Prospective multicenter studies with repeated urine testing, complete confounder adjustment and external validation are needed to confirm these associations and dissect its underlying biological relevance.

## Data Availability

The raw data supporting the conclusions of this article will be made available by the authors, without undue reservation.

## Supplementary material

Supplementary Table 1 Fitted linear regression model used to predict ln(UGE + 1) for construction of GB-UGE deficit. The dependent variable was ln(24-hour UGE [g/day] + 1). Model n = 887; R² = 0.3035; adjusted R² = 0.3003; RMSE = 1.1353; F(4,882) = 96.07, P < 0.001. The fitted equation was ln(UGE + 1) = -1.01114 + 0.14372 × HbA1c + 0.06479 × FPG + 0.06393 × 2hPG + 0.00056 × eGFR. GB-UGE deficit was defined as the negative residual and standardized within the analytic cohort. RMSE is reported on the ln(UGE + 1) scale.
