# Supplementary material for: Glycemic burden-adjusted urinary glucose excretion deficit is associated with hyperuricemia in patients with type 2 diabetes
Source: Front Endocrinol (Lausanne). 2026 Sep 18;17:1939076. doi: 10.3389/fendo.2026.1939076 (PMC13630666; doi:10.3389/fendo.2026.1939076)
Supplement: Supplementary file 1 [file DataSheet1.docx]

Supplementary Table 1. Fitted linear regression model used to predict ln(UGE + 1) for construction of GB-UGE deficit

| Predictor | β coefficient | Standard error | 95% CI | P value |
| --- | --- | --- | --- | --- |
| Intercept | -1.01114 | 0.18763 | -1.37940 to -0.64287 | <0.001 |
| HbA1c, % | 0.14372 | 0.02080 | 0.10290 to 0.18454 | <0.001 |
| FPG, mmol/L | 0.06479 | 0.01475 | 0.03584 to 0.09374 | <0.001 |
| 2hPG, mmol/L | 0.06393 | 0.00835 | 0.04755 to 0.08032 | <0.001 |
| eGFR, mL/min/1.73 m² | 0.00056 | 0.00079 | -0.00100 to 0.00212 | 0.484 |

*Note: The dependent variable was ln(24-hour UGE [g/day] + 1). Model n = 887; R² = 0.3035; adjusted R² = 0.3003; RMSE = 1.1353; F(4,882) = 96.07, P < 0.001. The fitted equation was ln(UGE + 1) = -1.01114 + 0.14372 × HbA1c + 0.06479 × FPG + 0.06393 × 2hPG + 0.00056 × eGFR. GB-UGE deficit was defined as the negative residual and standardized within the analytic cohort. RMSE is reported on the ln(UGE + 1) scale.*
